# Distribution Characteristics of Phosphorus in the Sediments and Overlying Water of Poyang Lake

**DOI:** 10.1371/journal.pone.0125859

**Published:** 2015-05-04

**Authors:** Lingqing Wang, Tao Liang

**Affiliations:** Key Laboratory of Land Surface Pattern and Simulation, Institute of Geographical Sciences and Natural Resources Research, Chinese Academy of Sciences, Beijing 100101, China; Peking UIniversity, CHINA

## Abstract

Phosphorus (P) is a key indicator of the aquatic organism growth and eutrophication in lakes. The distribution and speciation of P and its release characteristics from sediments were investigated by analyzing sediment and water samples collected during high flow and low flow periods. Results showed that the average concentrations (ranges) of total phosphorus (TP) in the surface and deep water were 0.06 mg L^-1^ (0.03–0.13 mg L^-1^) and 0.15 mg L^-1^ (0.06–0.33 mg L^-1^), respectively, while the average concentration (range) of TP in sediments was 709.17 mg kg^-1^ (544.76–932.11 mg kg^-1^). The concentrations of TP and different forms of P varied spatially in the surface sediments, displaying a decreasing trend from south to north. P also varied topographically from estuarine areas to lake areas. The vertical distribution of TP and different forms of P were observed to decrease as depth increased. The P concentrations during the low flow period were higher than those during the high flow period. Inorganic phosphorus (IP) was the dominant form of P, accounting for 61%–82% of TP. The concentration of bioavailable phosphorus in sediments was relatively large, indicating a high risk of release to overlying water. The simulation experiment of P release from sediments showed that the release was relatively fast in the first 0-5 min and then decreased to a plateau after 1 hr. Approximately 84–89% of the maximum amount of P was released during the first hour.

## Introduction

Eutrophication can result in significant deterioration in water quality due to the increased growth of undesirable algae and aquatic weeds followed by oxygen depletion due to biomass death and decomposition [1, 2]. Lake eutrophication has become a serious environmental problem in China, especially for shallow lakes in the middle and lower reaches of Yangtze River area [3, 4]. Poyang Lake is the largest freshwater lake in China as well as an important river-connected lake in the lower Yangtze region. Over the past several decades, an increase in nutrient inputs has led to persistent harmful algal blooms, which significantly degraded water quality of Poyang Lake [5].

Excessive concentrations of phosphorus (P) is the most common cause of eutrophication in freshwater lakes, reservoirs, streams, and in the headwaters of estuarine systems [6, 7]. The amount of phosphorus present in a water body depends on both the external phosphorus load and its release and retention in the sediments. Sediments act as a sink where P can be stored, and also as a source of P for the overlying water [8]. Recycling of P from sediments enriched by years of high nutrient inputs causes lakes to remain eutrophic even after external inputs of phosphorus are decreased [9]. Concern regarding the eutrophication of lakes has grown in recent years, leading to implementation of P reduction measures [10, 11, 12]. Although exogenous P loads have been reduced, endogenous release remains a key contributor to eutrophication [13, 14, 15]. Ongoing release and absorption of P between the sediments and overlying water create a dynamic balance in eutrophic water bodies. Thus, a better understanding of the form of P and its release from sediments would better inform pollution prevention measures for eutrophic waters.

To assess the risk of eutrophication in aquatic systems it is necessary to know not only the total P content in the sediments but also its fraction distribution among the different sediment phases. The forms of P present are dependent on physicochemical properties and migration and transformation processes at the lake sediment-water interface [16, 17]. Environmental factors can also alter the sediment properties by changing the physicochemical properties of overlying water, eventually influencing the deposition and release of P. Pertinent environmental factors including lake surface water temperature, pH, dissolved oxygen content, sediment-water exchange processes, and depth of the overlying water [7, 18, 19, 20]. Previous researchers have characterized the extraction, analysis, and release mechanisms of most forms of P [3, 4, 16, 21].

Poyang Lake is the largest freshwater lake in China as well as an important river-connected lake in the lower Yangtze region. The water level and water area are highly variable depending on season. In the recent years, eutrophication and water shortages were exacerbated as a result of climate change, regional economic development, and water storage related to the Three Gorges Project [22]. The rapidly changing hydrology and hydrodynamic conditions, as well as frequent flow and sediment flux exchange, make the mechanism of P cycling at the water-sediment interface more complex [23]. In this study, the spatial distribution, form, and release kinetics of P were systematically studied in order to characterize the biogeochemical cycling of P and the mechanisms of nutrient release from sediments of Poyang Lake. The results of this study could help better understand the P dynamics at the sediment—water interface in eutrophic lake.

## Materials and Methods

### Site Description

**Fig 1:**
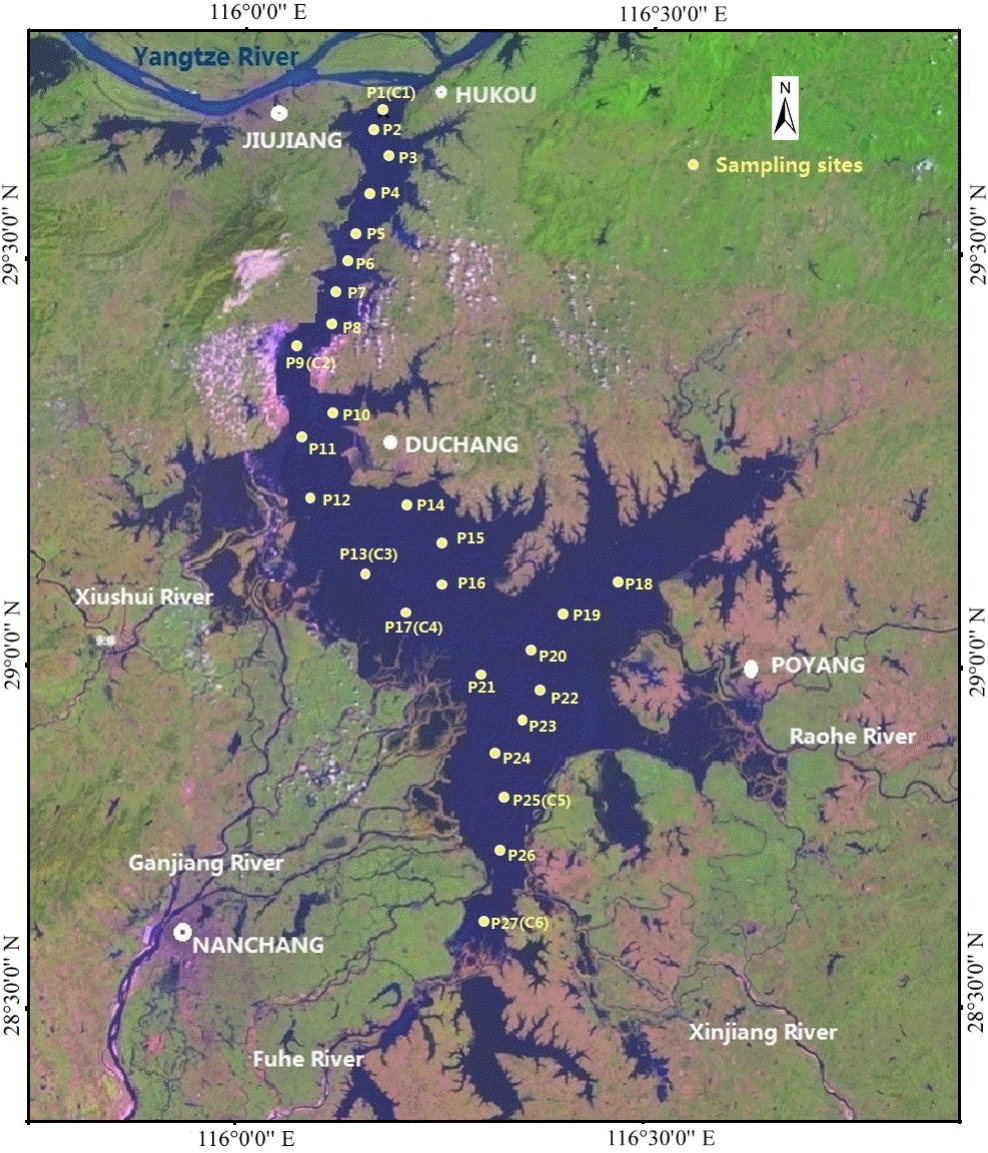
Map of the study area and sampling sites.

Poyang Lake is located on the south bank of the Yangtze River, in northern Jiangxi Province, China, at E115°49′–116°46′and N28°24′–29°46′. The lake receives water primarily from five rivers (the Gan, Fu, Xinjiang, Rao, and Xiu rivers) and provides regulation and storage capacity prior to their intersection with the larger Yangtze River through the lake outlet (Fig 1). Although the water level of Poyang Lake remains relatively high for long periods of time due to constant river inputs, the level can vary largely with alternating periods of floods and droughts. The area and volume of Poyang Lake also change considerably between the high flow and low flow periods. The lake area and volume are approximately 27 and 66 times larger during the wet season compared with the dry season [5]. The annual average water exchange cycle in Poyang Lake is 19 d and the average depth is 8.4 m, with the deepest point of the lake at ca. 25.1 m. The lake volume is ca. 27.6 billion m^3^, making Poyang Lake the largest freshwater lake in China.

The Poyang Lake basin is located in the East Asian Monsoon Region and has a subtropical warm and humid climate. The climate is mild with an average temperature of 17℃ and average annual precipitation of 1200–1700 mm. Generally, the flood season occurs during the last ten days of March, at which point flood water from the five rivers flows into the lake, causing the water level to rise and reach a peak in July. The water volume flowing into the lake from April through July accounts for 66.6% of the total volume annually, while the water volume flowing out during the same period accounts for 55.7%. The influence of flood intrusion during flooding season from July to September allows for a stable water level until October, at which point the water level begins to decrease gradually. The water level reaches its lowest point in January and February of the following year. Due to the rapid development of the Poyang Lake region, applications of fertilizers and pesticides, pollution discharge from towns, and population expansion, large amounts of nutrients enter the lake, exacerbating already serious eutrophication issues.

### Sample collection

The Poyang Lake basin climate is dominated by the Southeast Asian monsoon in summer, with a rainy season starting from April. During the high flow period from April to September, the floodplains are inundated and thus form a large lake with an area >3000 km^2^. During the low flow period from October to March, the lake's inundation area generally shrinks to <1000 km^2^, forming a narrow meandering channel. Water and sediment samples were collected in both the high flow and low flow periods across Lake Poyang and at the estuaries of the five rivers. No specific permissions were required for sampling activities in these locations. We confirmed that these field studies did not involve endangered or protected species. Twenty-seven sediment samples (labeled Pn, n = 1–27) were collected by a Van Veen Grab Sampler (0.5 L capacity). At each site, both surface and bottom water samples were collected (Fig 1). Six sediment cores (labeled Cn, n = 1–6) were collected using a gravity stainless steel sampler with a PVC tube. The top 20 cm of each core was sectioned at 1-cm intervals with a piston used to push out the mud column and metal fixed rings inserted to stratify the cores. The sediment and water samples were packaged immediately in polyethylene film and plastic sampling bottles immediately following collection. Both the sediment and water samples were stored in a cold incubator in the dark during transport to the laboratory.

### Measure and analysis

A portion of each sediment sample was freeze-dried using vacuum, purified, grind in agate mortar and then sieved through a 100-mesh sieve. The finished samples were saved in dryer. The water samples were analyzed immediately upon arrival to the lab. Sediment concentrations of different forms of P were determined by the SMT (Standard Measurements and Testing) protocol proposed by the European Commission [24], selected for its ease of use and reproducible results [4]. The protocol used is considered to be a harmonised protocol for P fractionation and P was separated into five fractions by this method. P bound to Al, Fe, and Mn oxides and oxyhydroxides was extracted by NaOH (Fe/Al–P). P bound to calcium was extracted by HCl (Ca–P). In a separated extraction, inorganic P (IP) was extracted by HCl and the residual was treated at 450°C to analyze organic P (OP). TP in the sediment samples was determined by processing the sample at 450°C, followed by HCl extraction. Detailed experimental conditions are described in the previous studies [3, 24]. All the samples were analyzed in triplicate, with the results being reported as the average values.

### Sediment P release experiment

Surface samples (0.25 g) of the six cores were combined with 25 mL of deionized water in centrifuge tubes and shaken (180~200 r·min^-1^) at indoor temperature (22±1°C) for 15, 30, 60, 90, 120, 150, 180, or 300 min. Samples were then centrifuged (4000 r·min^-1^) for 10 min and the supernatants were filtered through 0.45-μm filters and analyzed for phosphate via a molybdenum-antimony anti-spectrophotometric method [4, 8]. All treatments were carried out in triplicate and averaged with relative errors less than 5%.

### Calculations and statistics

All statistical analyses were conducted using SPSS 13.0 and the relationship between TP and different forms of P was evaluated using Pearson’s correlation. A global positioning system (GPS) recorded the locations of the samples and the geostatistical analysis was carried out with the extension Geostatistical Analyst of the GIS software ArcGIS (version 10.02). Origin 8.0 was used for creating the figures.

## Results and Discussion

### The spatial distribution of TP in Poyang Lake

**Fig 2:**
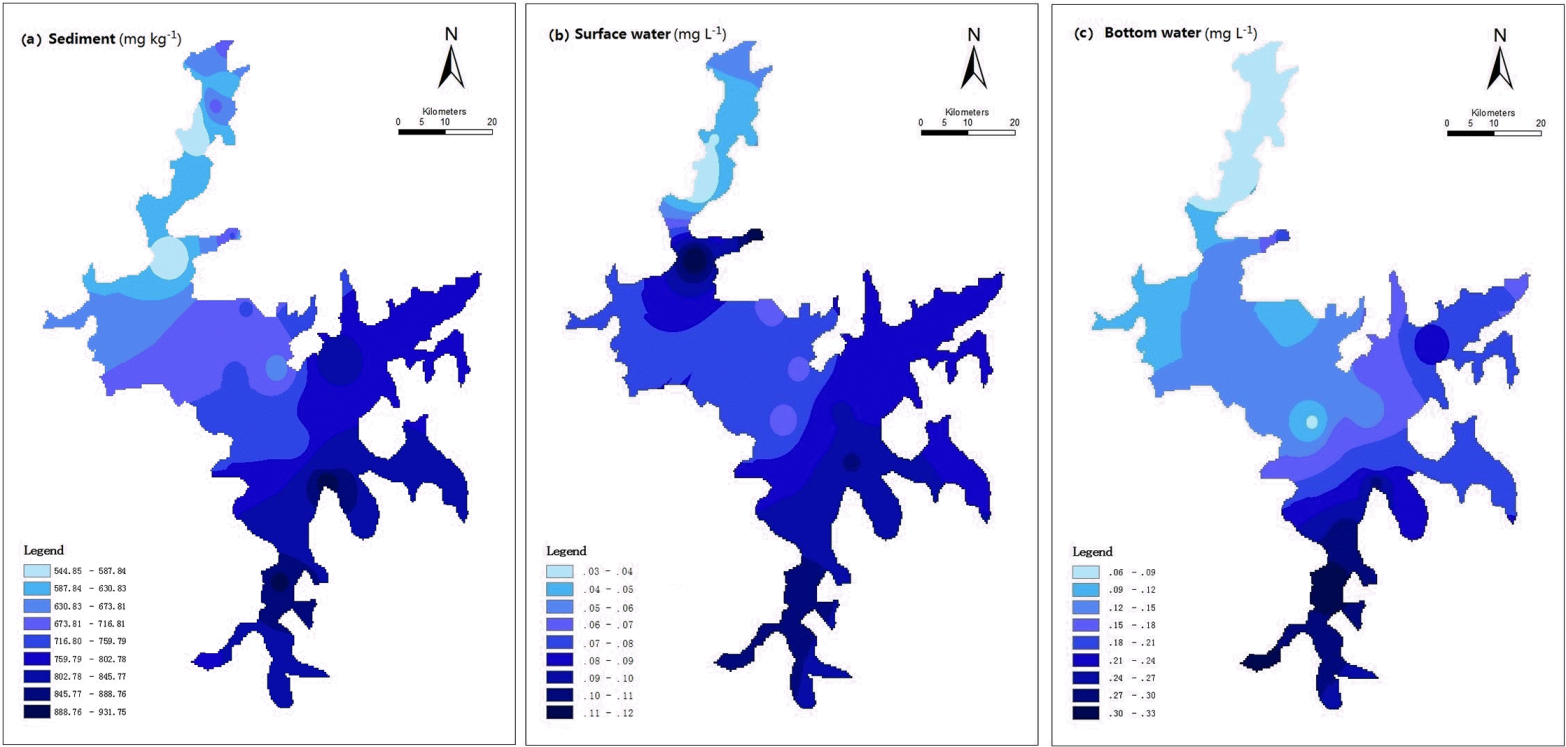
Spatial distributions of TP in the Poyang Lake.

The results of a one-way ANOVA showed significant spatial differences in TP among Poyang Lake sediment samples (P<0.01). The average concentration of TP was 709.17 mg kg^-1^ with a range of 544.76 and 932.11 mg kg^-1^ (Fig 2). The average concentrations (range) of TP in sediments during the low- and high-flow periods were 749.20 mg kg^-1^ (515.51–1051.90 mg kg^-1^) and 669.15 mg kg^-1^ (508.24–832.53 mg kg^-1^), respectively. The concentrations of TP were observed to decrease from east to west and from south to north, where TP at the inputs of the Rao and Xinjiang rivers was higher compared to other sites. The water velocity in the northern part of Poyang Lake was greater than that in the middle and southern regions of the lake. The faster velocities resulted in an increase in dilution, biodegradation, and deposition of P, leading to lower concentrations of P in the lake. Due to the influence from the inflows of the Gan, Xiu, and Boyang rivers, the concentration of P was higher in the lower stream, which can be attributed to industrial and municipal waste point sources.

The average concentration (range) of TP in surface water and deep water was 0.06 mg L^-1^ (0.03–0.13 mg L^-1^) and 0.15 mg L^-1^ (0.06–0.33 mg L^-1^), respectively (Fig 2). It is generally agreed upon that algal blooms occur in water bodies when the concentration of TP reaches 0.02 mg L^-1^ [27]. In this study, the TP concentrations were found to exceed this critical concentration. The spatial distribution of TP in the overlying water was similar to that in the sediment with a few notable differences. When the TP concentration was high in a region of sediment, corresponding high concentrations were measured in water, indicating that exchange and diffusion of P between sediment and water was occurring. Compared with the low water season, the TP concentration decreased greatly in high water season, this behavior can be attributed to higher water temperatures, greater biologic activity, and algal blooms which promote the depletion of dissolved P.

### The variation characteristics of different forms of P in sediment

**Fig 3:**
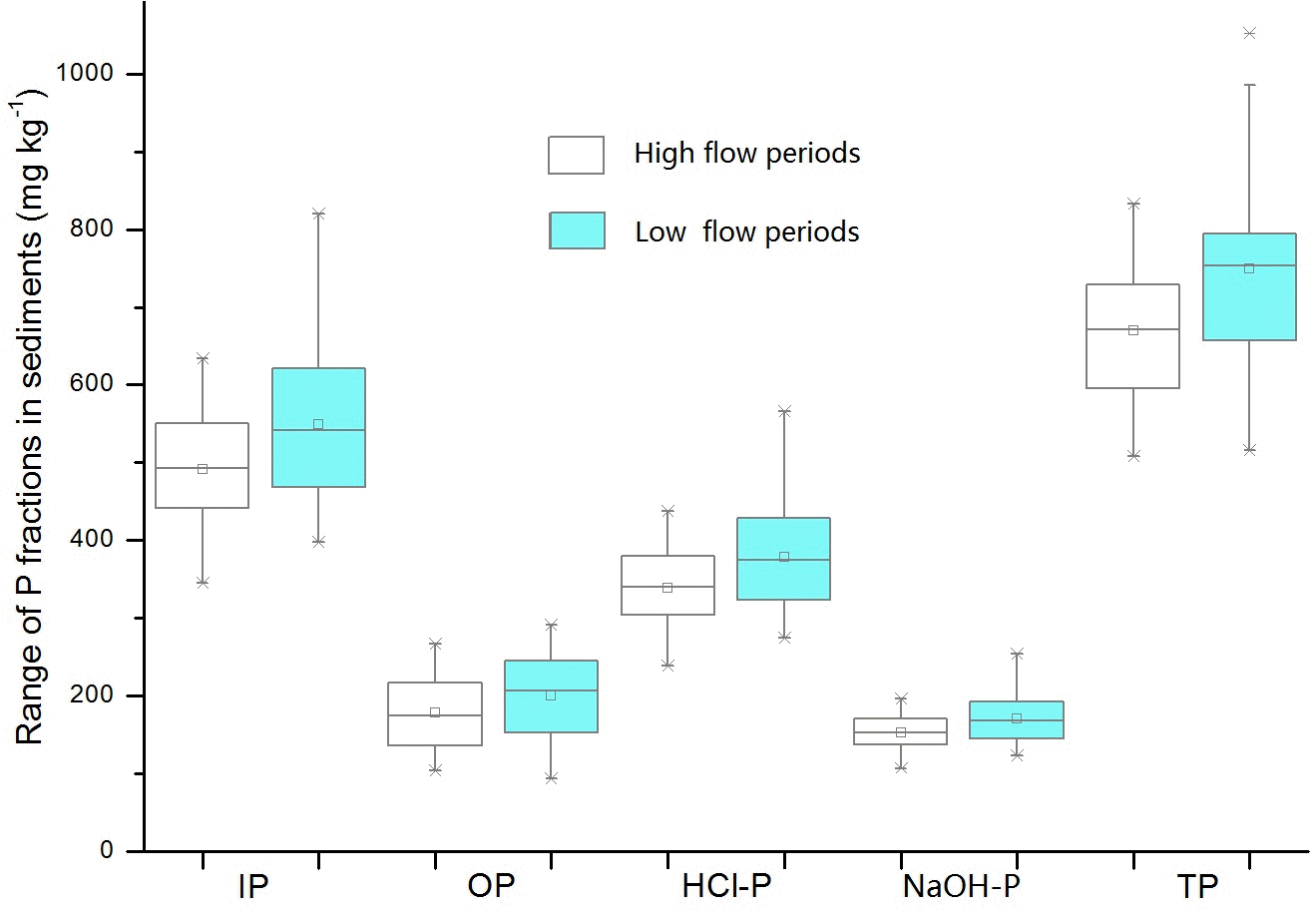
Concentrations of P fractions in sediments in high flow and low flow periods.

The concentrations of different forms of P varied significantly among different sample sites and illustrated the effects of human activity on the lake (Fig 3). The average concentration (range) of OP in sediments was 178.16 mg kg^-1^ (103.38–266.41 mg kg^-1^) in the high flow period and 199.81 mg kg^-1^ (92.79–290.92 mg kg^-1^) in the low flow period, accounting for 18%-38% of TP. The concentrations of OP in sediments were low. OP is released only when organic matter in the lake is mineralized [3, 26, 28]. Eutrophication in Poyang Lake caused an increase in the amount of organic matter originating from bacteria and algal sources. Cyanobacteria, which bloom during the high water season, significantly increase the OP value when they die and decompose [27, 28].

In the SMT method, IP, including HCl-P and NaOH-P, indicates the presence of P in different forms by combining dissolved P absorbed on sediments with metal ions in water. The average concentration of IP in Poyang Lake sediments was 490.99 mg kg^-1^ in wet season and 549.38 mg kg^-1^ in dry season, accounting for 61%-82% of TP and showing that IP accounts for the majority of P in sediments.

The NaOH-P separated by the SMT method is Fe/Al-P and primarily composed of P that can combine with metal oxides, such as iron and aluminum oxides, and exchange with OH^-1^ and organic ligands [3, 24, 25, 26]. These forms of P are readily dissolved under alkaline conditions and converted into dissolved P when the redox environment changes [12, 25]. Dissolved P can then enter the overlying water, and finally worsening the water quality. The average concentration (range) of NaOH-P in Poyang Lake sediments was 338.79 mg kg^-1^ (238.47–437.17 mg kg^-1^) in wet season and 379.08 mg kg^-1^ (274.28–566.13 mg kg^-1^) in dry season, accounting for 35%-75% of IP and showing that NaOH-P accounts for the majority of IP in sediments.

HCl-P cannot be easily used by organisms or released into overlying water. HCl-P is mainly derived from clastic rock or in situ synthesis and is not considerably affected by human activities [15, 27, 30, 31]. The average concentration of HCl-P in Poyang Lake sediments was 152.21 mg kg^-1^ in wet season and 170.31 mg kg^-1^ in dry season, accounting for 18%-25% of IP. The concentration was found to increase with an increase in TP concentration and accounted for 7%-29% of TP.

In general, NaOH-P and OP can release bioavailable forms of P which can potentially be used directly or indirectly by algae [4, 33]. The concentration of bioavailable P reflects the degree of pollution and the endogenous release ability. Bioavailable P can be transformed into active P through chemical and biological reactions and in turn influence the overlying water quality [30]. Several studies have shown that higher amounts of bioavailable P in sediments result in greater release of P [10, 29, 30, 33]. Based on research by Rydin [35], approximately 50–60% of OP in sediments can be degraded or hydrolyzed into bioavailable P. In this study, both the concentration and the proportion of bioavailable P were considerable, indicating that the potential availability and release risk to the overlying water were high.

The results of the correlation analysis showed a significant positive correlation between TP content and IP, OP, HCl-P, and NaOH-P content in Poyang Lake sediments. An apparent positive correlation between IP content and HCl-P and NaOH-P contents was also found (Table 1), suggesting that the increase in TP and simultaneous release of P from sediment enhanced the availability and release risk of P.

**Table 1:** Pearson's correlations between TP and P fractions in the surface sediments (n = 27, p<0.05).

	TP	IP	OP	NaOH-P	HCl-P
TP	1				
IP	0.89	1			
OP	0.55	0.13	1		
NaOH-P	0.87	0.99	0.09	1	
HCl-P	0.90	0.96	0.23	0.93	1

### The vertical distribution of TP and forms of P in sediments

**Fig 4:**
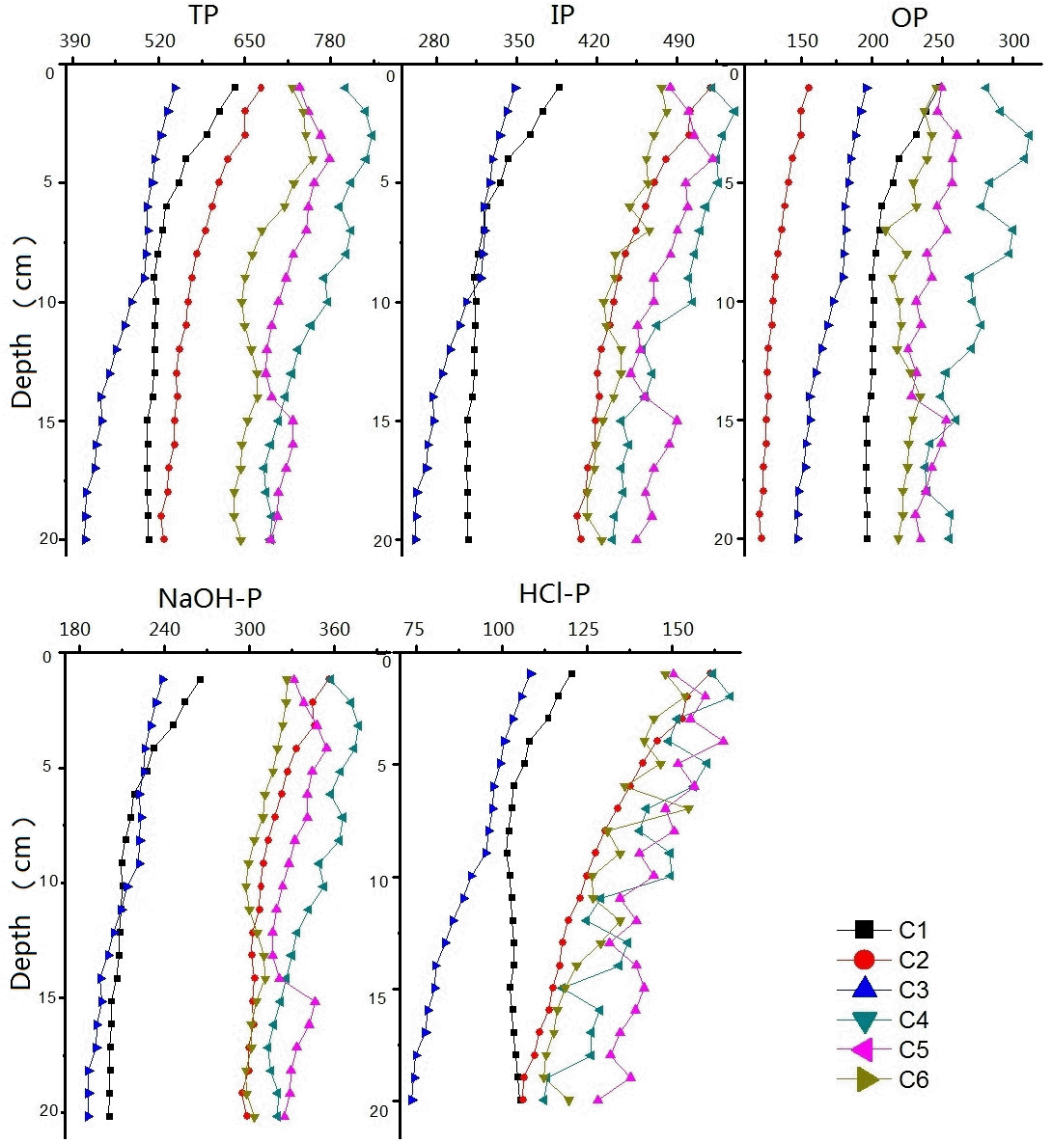
Vertical distribution of P fractions in sediment cores of Poyang Lake.

The vertical distribution of different forms of P in lake sediments may indicate various migration and transformation patterns. OP and NaOH-P are converted into IP and HCl-P, respectively, following the anaerobic release of P from sediments [30, 31, 34]. The vertical distribution of TP and various forms of P decreased vertically with increasing depth up to 15 cm. The average concentration in the sediment surface layer was higher than that in bottom, suggesting an enrichment of P may be attributed by the input of external sources (Fig 4). The distribution of TP showed a clear increase in concentrations of TP and different forms of P in recent years.

**Fig 5:**
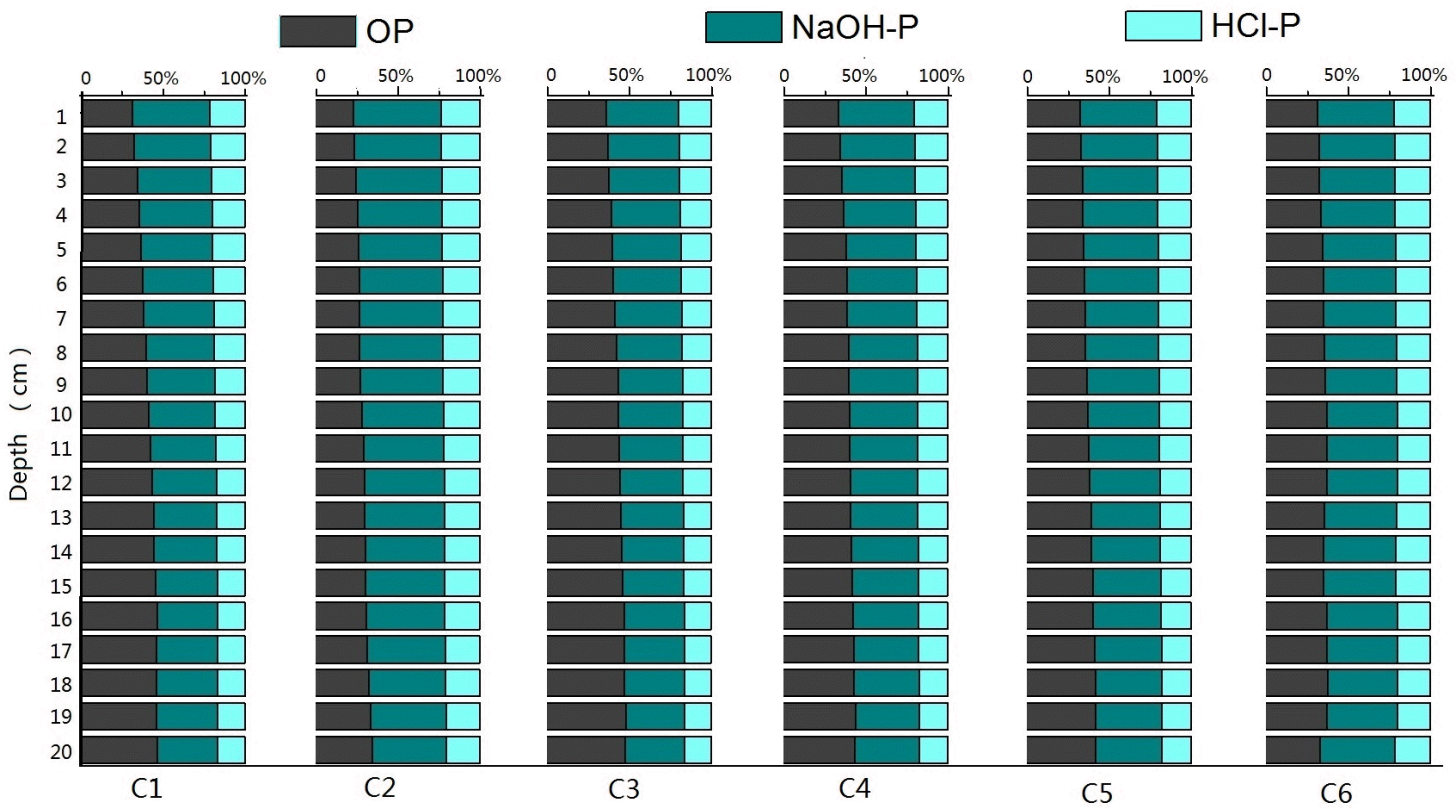
The percentage of P fractions in sediment cores of Poyang Lake.

The vertical distribution of average concentrations of TP and different forms of P was found to vary with location. As shown in Fig 5, the contents of OP in sediment cores were lower than that of IP in the upper layers of six sediment cores. The average percentages (range) of OP, NaOH-P and HCl-P in sediment cores were 37% (23%~46%), 44% (35%~53%) and 19% (16%~24%), respectively. Both OP and IP in sediments may be transferred to each other and released into the overlying water during mineralization, which results in considerable changes in the phosphorus distributions [29, 31, 33]. HCl–P was considered to be related to the local geology, while OP was considered to be primarily from rural agricultural cultivation and fertilization and was a steady, but continuously bio-available fraction of phosphorus [19, 27, 33]. Thus, according to the analysis of phosphorus fractions in sediment cores, the primary sources of pollution can be assessed and various restoration measures can be adopted for different regions.

The average concentrations of TP and various forms of P in cores C4, C5, and C6 collected from estuaries were higher than those in cores C1, C2, and C3 collected in the center of the lake and in the main channel areas (far away from estuary). The vertical distribution of TP and various forms of P also covered a smaller range in C1, C2, and C3 than in C4, C5 and C6, possibly attributed to more effective hydrodynamic exchange in estuary water (C4, C5 and C6). Hydrodynamics may result in large disturbances to the sediments and intensify P exchange and diffusion at the sediment-water interface [25, 26, 36, 37].

### The release characteristics of P in sediments

**Fig 6:**
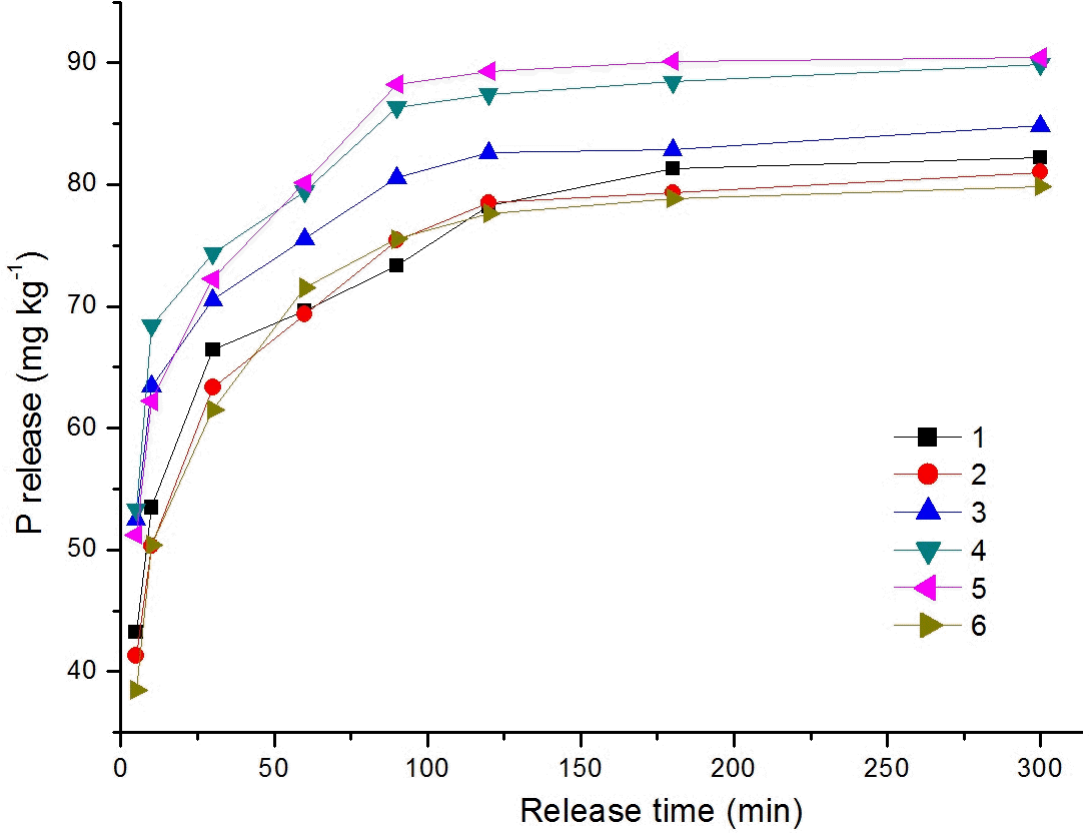
Release kinetic curve of P in the surface sediments of Poyang Lake.

The maximum phosphorus release from sediments ranged from 79.84–90.42 mg kg^-1^, with an average value of 84.71 mg kg^-1^. The maximum release rate was positively correlated with the concentration of TP in surface sediments of Poyang Lake (R = 0.48, n = 6, p<0.05). The release amount increased as TP concentration increased, with regions containing high TP concentrations displaying the largest release amounts. The release rate was relatively fast in the first 60 min and then gradually slowed (Fig 6). The dynamic equilibrium between the absorption and release of P at the sediment-water interface is characterized as P cycling in the lake. In general, P in the sediments is only released when the concentration of P in the overlying water decreases. Results from this study show that P can also be released from sediments, creating a dynamic balance of P at the sediment-water interface. The absorption and release of P are dependent not only on the phosphate concentration in water but also on environmental conditions [21, 29, 37, 38]. When the environment changes, the dynamic balance is disturbed and P can be released into water through a number of complex physical and chemical processes causing secondary pollution [32, 33, 39, 40].

The release rate of P (i.e., the release amount of P from unit mass sediments per unit time) was calculated in Poyang Lake sediments. As shown in Table 2, the release rate in the first 0–5 min was the highest, varying from 8.26–10.66 mg (kg·min)^-1^, and accounting for 48–61% of the maximum release amount. The release rate decreased gradually after 5 min and was less than 1 after 60 min. The release amount in the first 60 min accounted for approximately 84–89% of the maximum value. The release rate of P in the first 0–5 min was positively correlated with the TP concentration in sediments.

**Table 2:** The release rate of P in surface sediments of Poyang Lake in different intervals.

Time/min	Release rate mg (kg·min)^-1^					
	1	2	3	4	5	6
0–5 min	8.65	8.26	10.50	10.66	10.25	7.70
>5–10 min	5.35	5.03	6.34	6.84	6.22	5.04
>10–30 min	2.21	2.11	2.35	2.48	2.41	2.05
>30–60 min	1.16	1.16	1.26	1.32	1.34	1.19
>60–90 min	0.81	0.84	0.89	0.96	0.98	0.84
>90–120 min	0.65	0.65	0.69	0.73	0.74	0.65
>120–180 min	0.45	0.44	0.46	0.49	0.50	0.44
>180–300 min	0.27	0.27	0.28	0.30	0.30	0.27

## Conclusions

The average concentration (range) of TP in sediments was 709.17 mg kg^-1^ (544.76–932.11 mg kg^-1^). During the high flow period, the average concentration (range) of TP was 749.20 mg kg^-1^ (515.51–1051.90 mg kg^-1^) compared to 669.15 mg kg^-1^ (508.24–832.53 mg kg^-1^) during the low flow period. The concentration of TP was found to decrease in magnitude from east to west and from south to north. The average concentrations of TP in surface and deep water samples were 0.06 mg L^-1^ (0.03–0.13 mg L-1) and 0.15 mg L^-1^ (0.06–0.33 mg L^-1^), relatively. The concentration of P reached and exceeded the critical concentration of 0.02 mg L^-1^. The average concentrations of TP and various forms of P in six cores decreased as depth increased. The IP accounted for 61%-82% (mass fraction) of TP, showing that IP accounts for the majority of P in sediments. OP accounted for 18%-38% (mass fraction) of TP. NaOH-P accounted for the majority of IP (35%-75%) and HCl-P accounted for 18%-25% of IP. Rapid release of P occurred in the 0–5 min after which the rate gradually decreased and reached a balance. The release rate of P was positively correlated with the TP concentration in sediments, showing that the release rate of P from sediments increases with the increase of TP concentration and that regions with high TP concentrations release greater amounts of P.

## Supporting Information

S1 Table Poyang Lake Data. (XLSX)
